# Inflammation and Severe Cerebral Venous Thrombosis

**DOI:** 10.3389/fneur.2022.873802

**Published:** 2022-07-22

**Authors:** Shuyuan Hu, Hangil Lee, Haiping Zhao, Yuchuan Ding, Jiangang Duan

**Affiliations:** ^1^Department of Emergency, Xuanwu Hospital, Capital Medical University, Beijing, China; ^2^Department of Neurology and Intracranial Hypertension and Cerebral Venous Disease Center, National Health Commission of the People's Republic of China, Xuanwu Hospital, Capital Medical University, Beijing, China; ^3^Department of Neurosurgery, Wayne State University School of Medicine, Detroit, MI, United States; ^4^Cerebrovascular Diseases Research Institute and Department of Neurology, Xuanwu Hospital, Capital Medical University, Beijing, China

**Keywords:** cerebral venous thrombosis, cerebral venous infarction, inflammation, pathophysiology, blood-brain barrier

## Abstract

Cerebral venous thrombosis (CVT) is a rare type of venous thromboembolism (VTE). It is an important cause of stroke in young adults and children. Severe CVT, which is characterized by cerebral venous infarction or hemorrhage, seizures, or disturbance of consciousness, has more severe clinical manifestations and a worse prognosis. It is commonly believed that the onset of severe CVT gave credit to venous return disorder, with the underlying pathogenesis remaining unclear. There is increasing evidence suggesting that an inflammatory response is closely associated with the pathophysiology of severe CVT. Preclinical studies have identified the components of neuroinflammation, including microglia, astrocytes, and neutrophils. After CVT occurrence, microglia are activated and secrete cytokines (e.g., interleukin-1β and tumor necrosis factor-α), which result in a series of brain injuries, including blood-brain barrier disruption, brain edema, and cerebral venous infarction. Additionally, astrocytes are activated at the initial CVT stage and may interact with microglia to exacerbate the inflammatory response. The extent of cerebral edema and neutrophil recruitment increases temporally in the acute phase. Further, there are also changes in the morphology of inflammatory cells, expression of inflammatory mediators, and inflammatory pathway molecules with CVT progression. Lately, some clinical research suggested that some inflammation-related biomarkers are of great value in assessing the course, severity, and prognosis of severe CVT. Moreover, basic and clinical research suggested that anti-inflammatory therapy might hold promise in severe CVT. This study reviews the current literature regarding the involvement of inflammation in the pathophysiology and anti-inflammatory interventions of severe CVT, which would contribute to informing the pathophysiology mechanism and laying a foundation for exploring novel severe CVT therapeutic strategies.

## Highlights

- Inflammatory reactions are crucially involved in the development of acute/subacute severe CVT and are related to poor prognosis.
- Inflammatory cells, mediators, and signaling pathways are involved in severe CVT brain injury.
- Cerebral venous infarction and inflammatory response have a mutually causal relationship.
- Inflammation-sensitive biomarkers could be used for staging and prognostication of severe CVT.
- Anti-inflammation is a promising therapeutic modality for acute/subacute severe CVT.

## Introduction

Cerebral venous thrombosis (CVT) is a rare type of venous thromboembolism (VTE) and an important cause of stroke in young adults and children (1), with approximately 75% and <10% of patients with CVT being aged 16–50 and >65 years (2, 3). Recent population-based studies have reported that the annual incidences of CVT among adults in Finland and Australia are 1.32 and 1.57 per 100,000, respectively (4, 5). The incidences of CVT in Asia and the Middle East may be even higher given the higher incidences of pregnancy and infection (6). Large cohort studies have demonstrated favorable long-term outcomes in most patients with CVT (7–9). However, approximately 13% of all patients with CVT die or remain handicapped (modified Rankin Scale [mRS] >2) (7). Some patients with CVT develop cerebral venous infarction/hemorrhage, seizures, mental status impairment, disturbance of consciousness (Glasgow scale score <9), and straight sinus thrombosis, which are the characteristic features of severe CVT that lead to more severe clinical manifestations and worse prognosis (10–13).

It is important to elucidate the pathophysiology underlying severe CVT for improved treatment and prognostication. Traditionally, the onset of severe CVT was described as a venous return disorder, with the underlying pathogenesis remaining unclear (14). A recent clinical trial demonstrated that endovascular treatment, which can improve venous return disorder, could not improve functional outcomes in patients with CVT; accordingly, it was terminated early for futility (15). There is increasing evidence suggesting that the inflammatory response is crucially involved in regulating severe CVT pathogenesis and is strongly associated with poor prognosis (16, 17). Microglia (18, 19), astrocytes (18, 20, 21), and neutrophils (22) work jointly in the pathophysiology of severe CVT. Taken together, there could be a close relationship between inflammation and severe CVT, which could further inform the elucidation of the pathophysiology of severe CVT.

We aimed to review the current literature regarding inflammation in the pathogenesis of severe CVT, including the involvement of critical inflammatory cells, inflammatory mediators, and related inflammatory molecular signaling to further elucidate the relationship between inflammation and severe CVT.

## Correlation Between Inflammation and Severe Cerebral Venous Thrombosis

Inflammation is an essential response of the immune system that maintains body homeostasis (23). After immune cells migrate to the central nervous system (CNS), they interact with CNS resident cells through immune mediators to elicit immune cell responses (24, 25). After CVT onset, an inflammatory response occurs that is characterized by the activation of inflammatory cells and the release of inflammatory mediators. The intracellular inflammatory pathways of proinflammatory microglia are activated, which induce the release of numerous pro-inflammatory factors, including various cytokines and chemokines (18), with accompanying adhesion of circulating neutrophils (22). Taken together, these effects eventually result in a series of brain injuries, including blood-brain barrier (BBB) disruption, brain edema, and venous infarction, which lead to poor outcomes in patients with severe CVT (18, 22). Inconsistent with the notion that the inflammatory response is the catalyst for cerebral venous infarction, Rashad et al. (18) observed BBB disruption before inflammatory changes in rats with severe CVT. The delay between BBB breakdown and inflammatory changes after severe CVT suggests that molecules, including blood components and inflammatory factors, are leaked through the disrupted BBB to trigger neuronal injury and neuroinflammation (18, 26). Therefore, BBB disruption could be considered responsible for the inflammatory response in cerebral venous infarction. Accordingly, in severe CVT, there might be a mutually causal relationship between inflammatory response and BBB disruption.

**Figure 1:**
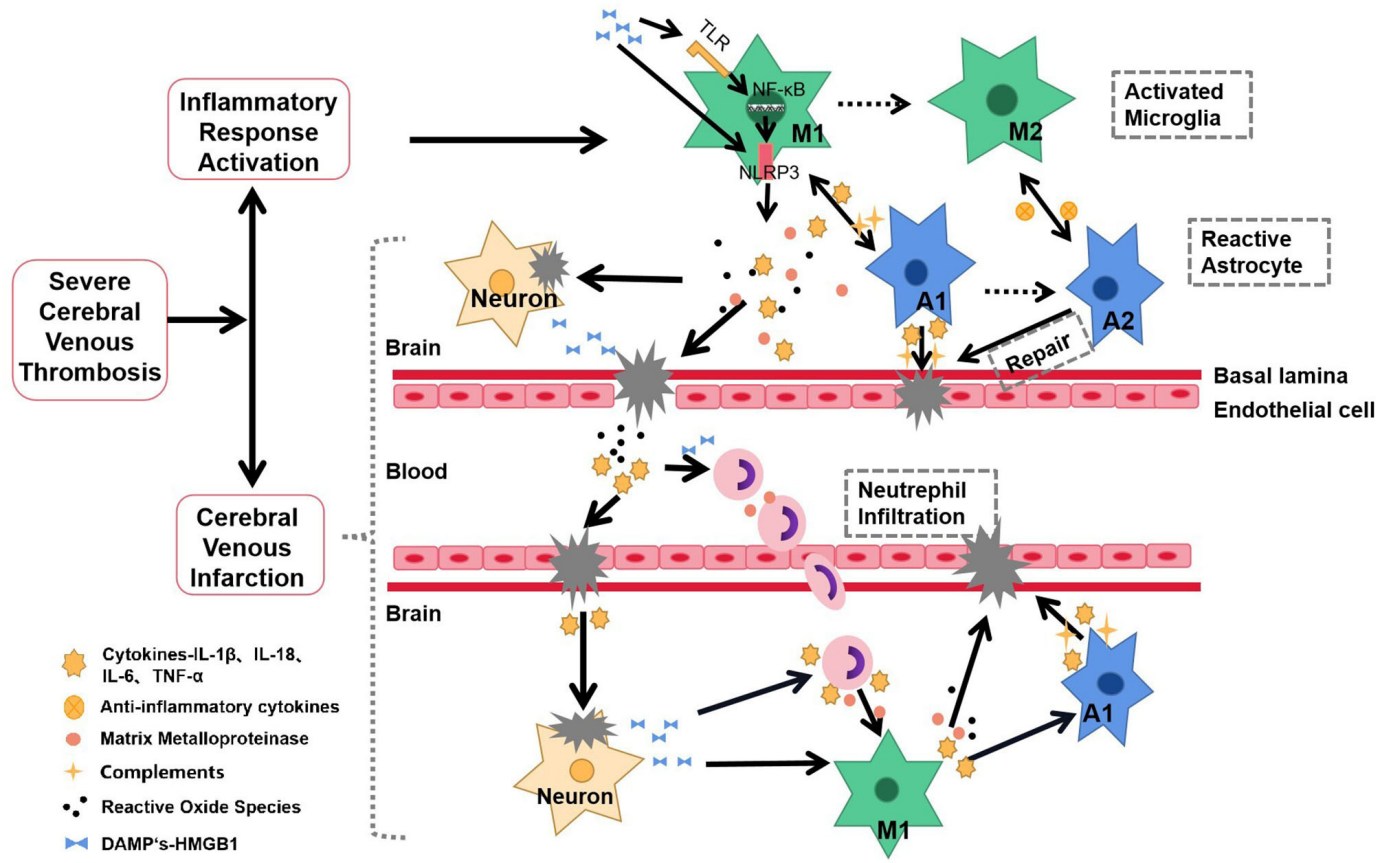
Schematic representation of inflammatory response in the pathophysiology and pathogenesis of severe CVT. After the onset of severe CVT, the inflammatory response is rapidly activated. The specific manifestations are as follows: central microglia and astrocytes are activated, presenting a proinflammatory state; secretion of inflammatory factors, MMPs, ROS, and other mediators lead to nerve injury, BBB destruction, and cerebral venous infarction. These proinflammatory factors also recruit neutrophils from the periphery into CNS, allowing the further release of proinflammatory mediators, which in turn activate additional microglia and astrocytes. Dying neurons release danger-associated molecular patterns (DAMPs), which activate NLRP3 inflammasomes through TLR-NF-κB signaling, which activates inflammatory cells to amplify the cascade of disease and injury. The inflammatory response forms a positive feedback loop with nerve injury, aggravating the incidence and intensity of cerebral venous infarction. Solid line, Based on studies with the available evidence; Dotted line, hypotheses that require further experimentation.

Cell death occurs with CVT progression (18), which leads to the release of danger-associated molecular patterns (DAMPs) (27). DAMPs comprise various altered metabolic products, including uric acid, mtDNA, S100, and heat shock proteins (HSPs), which are released by dying cells (28). DAMPs can be recognized by germline-encoded pattern recognition receptors (PRRs), including Toll-like receptor (TLR) and NLRP3, which activate downstream inflammatory signaling pathways to further aggravate inflammatory damages and amplify local inflammatory reactions (18, 22, 27, 29) (Figure 1). However, it remains unclear whether inflammation is involved in the subsequent repair of severe CVT.

### Activation of Inflammatory Cells

#### Microglia

Microglia are centrally involved in neuroinflammation. They belong to the mononuclear phagocyte lineage and are CNS-resident cells. Microglia usually present a ramified morphology in the normal brain, regulate brain development, maintain neuronal networks, and repair damage (30, 31). Inflammation, ischemia, and alterations in brain homeostasis can cause dramatic changes in the morphology, gene expression, and functional behavior of microglia, which is termed “microglial activation (32).” Microglial activation can be classified as classically or alternatively activated type (M1 or M2 type, respectively). The M1 type is characterized by the production of proinflammatory cytokines, including interleukin-1β (IL-1β), IL-6, tumor necrosis factor-α (TNF-α), and C-C chemokine ligand 2, which exacerbate the inflammatory response. Contrastingly, the M2 type is characterized by the production of cytokines, such as IL-10, transforming growth factor-β, IL-4, IL-13, and insulin-like growth factor-1, which suppress inflammation and promote tissue repair (33).

Moreover, the microglia-mediated inflammatory cascade is rapidly activated after the onset of severe CVT. Specifically, immediately after CVT onset, endoplasmic reticulum (ER) oxidative stress can be detected as IRE1α by activated microglia, which promotes programmed neuronal death. This indicates that microglia are involved in the early pathophysiology of severe CVT (19). Rashad et al. (18) observed extensive microglial infiltration in the superior sagittal sinus as early as 1 day after severe CVT. On the third day of cerebral venous infarction, microglia infiltration peaked and extended to the subarachnoid space and infarcted cortex. Moreover, microglia showed proinflammatory morphology, with an amoeboid shape rather than the usual ramifications, which is suggestive of M1 microglia.

Following CVT-induced disruption of cerebral blood flow, the adhesion of circulating neutrophils (22) and the release of inflammatory media, including cytokines and chemokines, ultimately induce a series of brain injuries, including BBB disruption, brain edema, and venous infarction (18–20). Activated microglia produce IL, TNF, chemokines, and other inflammatory mediators involved in inflammation regulation; further, they produce cytotoxic substances, including reactive oxygen species, to aggravate brain tissue injury (34, 35). Taken together, microglial activation may be significantly involved in the pathophysiology of severe CVT; however, its specific role and the phenotypic evolution of M1 and M2 remain unclear.

#### Astrocytes

Studies on innate inflammatory responses in the CNS have mainly focused on microglia, which are the CNS macrophages. The contribution of other cell types to inflammatory responses has been overlooked for a long time. However, there is emerging evidence indicating that astrocytes are actively involved in the brain's inflammatory response, with complex biphasic roles in the local regulation of inflammatory responses. Astrocytes, which are the most abundant glial cells of the CNS, originate from the neuroectoderm and are essential BBB components. They are crucially involved in maintaining brain homeostasis and neuronal function (36).

Similar to microglia, reactive astrocytes have two polarization states (the proinflammatory phenotype [A1] and anti-inflammatory phenotype [A2]), which both interact with microglia. M1 microglia activate A1 astrocytes by secreting factors such as IL-1 and TNF-α to set off an inflammatory response amplifying cascade (36). Upon activation, A1 astrocytes increase the expression of numerous genes of the classic complement cascade and inflammatory factors that communicate with M1 microglia and damage neural cells and the BBB (36, 37). Additionally, M2 microglia and A2 astrocytes work together to produce anti-inflammatory cytokines, including IL-6, IL-10, and cardiotrophin-like cytokine factor 1. A2 astrocytes promote BBB remodeling through the aforementioned beneficial cytokines. Specifically, interactions between M2-type microglia and A2-type astrocytes promote neuronal survival and repair (36).

On the third day after the onset of severe CVT in rats, there is dense staining of glial fibrillary acidic protein, which is specifically expressed by astrocytes, in the outer area of the venous infarction. This suggests that astrocytes are strongly activated and recruited in the cerebral venous infarct area, which is consistent with the region of microglia expression. This indicates that astrocytes are also involved in the initial stage of severe CVT (18). This is further indicated by reports of astrocyte infiltration in brain tissues with infarction or severe cerebral edema (19, 38). Astrocyte expression peaks on day 7 after severe CVT, with accompanying glial scars, which suggests that astrocytes may be involved in repair after cerebral venous infarction (18, 20). Furthermore, a clinical case report described that a patient who had isolated cortical venous thrombosis with severe seizures presented parenchymal destruction accompanied by reactive astrocyte proliferation in the excised lobar lesions (21). Taken together, besides microglia, astrocytes may be also actively involved in the inflammatory processes of severe CVT and mediate subsequent neural repair.

#### Neutrophils

Neutrophils are the earliest peripheral leukocytes recruited to the CNS after brain injury (34, 39). Additionally, they can respond to DAMPs and upregulate adhesion receptors after activation by inflammatory mediators such as TNF-α and interferon-gamma (INF-γ), which promotes their adhesion to endothelial cells and migration into inflammatory tissues. Neutrophil recruitment can enhance CNS damage by releasing lysozymes and secreting inflammatory mediators (39), which further activate microglia (26). Additionally, neutrophils can produce neutrophil extracellular traps, extracellular DNA fibers containing histones, and neutrophil antibacterial proteins, which promote the formation of venous thrombosis through various effects (40).

Eosinophilic necrotic foci and scattered neutrophils are commonly observed in the brains of rats with acute severe CVT (41). Additionally, brain water content and BBB permeability after severe CVT could be dependent on leukocyte-endothelial cell adhesion, with both showing a temporal increase during the acute phase. Although the mechanism underlying the relationship between leukocyte-endothelial cell adhesion and brain edema remains unclear, post-CVT brain edema and BBB dysfunction can be prevented by inducing neutrophil reduction using a CD-18 antibody (22).

A retrospective study on inflammatory markers and temporal changes in CVT suggested that compared with patients with chronic CVT, those with acute and subacute CVT showed a higher rate of adverse clinical outcomes and higher absolute neutrophil counts in the peripheral blood (42). Therefore, neutrophil regulation in early-stage CVT could be a potential therapeutic target.

### Inflammatory Mediators

Immune cells release substances, including cytokines, which are termed immune mediators and are crucially involved in inflammatory events. Cytokines act as signaling molecules that regulate different cellular functions and immune balance; moreover, they contribute to cell signaling and communication (43).

#### Interleukin Family

The interleukin-1 cytokine family is comprised of 11 proteins. They bind to their respective ligands and initiate inflammatory signaling and regulate immune response; among them, IL-1β and IL-18 act as the proinflammatory family members (44). IL-1β and IL-18 are closely associated with venous thrombosis (45, 46). Specifically, IL-1β is a key accelerator of venous thrombo-inflammation, which promotes venous thrombosis through several mechanisms, including leukocyte recruitment, remote signaling through thrombogenic microparticles, and platelet integrin activation (45). IL-1β and IL-18 are indicators of microglial and inflammasome activation, which intensifies the CVT severity (18). There are increased IL-1β (18, 19, 47) and IL-18 (18, 19) levels in the area of cerebral venous infarction in rats with acute/subacute severe CVT. IL-1β begins modestly increasing as early as 6 h after CVT, peaking on day 3 (18, 19) and remaining at a high level until day 7, followed by a return to baseline levels by day 14 (19). The trends of IL-18 and IL-1β expression are similar (18). After severe CT, immunofluorescence staining reveals significant microglial IL-1β levels (18), which further illustrate the inflammatory role of microglia in severe CVT.

Interleukin-6 can be produced by various cells, including monocytes, macrophages, endothelial cells, adipocytes, and the Th-2 subset of T-helper cells. IL-6 can transmit signals to endothelial cells, leukocytes, and hepatocytes; promote the synthesis of the coagulation factors (e.g., fibrinogen, tissue factor, and factor VIII); and stimulate platelet production. Specifically, IL-6 can mediate the coagulation cascade through various actions, which are closely associated with arteriovenous thromboses (48). An exploratory study on the acute CVT animal model reported significantly increased levels of proinflammatory cytokines, including IL-6 and IL-1β, compared with the sham group. Upon reduction of the levels of the aforementioned proinflammatory factors, there was a significant reduction in the cerebral venous infarct volume, which suggests that inhibiting inflammation may improve outcomes in severe CVT (47).

Notably, IL-6 can promote C-reactive protein (CRP) production by hepatocytes. IL-6 and plasma CRP levels increase during acute inflammation, which synergistically acts during the acute inflammatory phase. They are among the most commonly tested biomarkers for acute inflammation in clinical practice (49). Several studies have reported significantly higher serum IL-6 (17, 50) and Hs-CRP (17, 51)/CRP (52) levels in patients with severe CVT than in healthy controls, with IL-6 levels peaking on the first day of CVT diagnosis (50). This suggests that IL-6 and Hs-CRP levels are strikingly increased during the acute and subacute stages of CVT, followed by a decrease in the chronic phase (17, 51).

Contrastingly, another study reported no significant increase in the levels of pro-inflammatory factors, including IL-6, in patients with a history of CVT (53). This further indicates that inflammation occurs soon after the onset of severe CVT and is mainly involved in the acute/subacute phase. Additionally, IL-6 levels are positively associated with the risk of adverse prognostic events (17). This demonstrates that inflammatory factors are associated with severe clinical manifestations of CVT; moreover, they may be used as diagnostic biomarkers with predictive utility in the clinical treatment of severe CVT.

#### Tumor Necrosis Factor (TNF-α)

Tumor necrosis factor-α is a common proinflammatory factor involved in coagulation. TNF-α can regulate thrombogenic proteins, activate complements, and stimulate endothelial cells and macrophages to produce tissue factors, which ultimately promote coagulation (54). TNF-α is mainly produced by microglia in the CNS. In animal models of ischemic stroke, TNF-α-induced apoptosis of endothelial cells resulted in BBB disruption (55). Similarly, there was increased TNF-α expression in the area of cerebral venous infarction in rats with early-stage CVT. Notably, TNF-α expression is substantially reduced by recombinant human soluble thrombomodulin (rhs-TM), which inhibits the pro-inflammatory factors to prevent further brain damage in rats, and therefore, improves adverse outcomes (47).

#### High Mobility GroupBox-1 (HMGB1)

The high mobility groupbox-1 is a ubiquitous nuclear protein that maintains nucleosome integrity and promotes gene transcription; moreover, it is a DAMP protein. It can be released by granulocytes or necrotic cells into the extracellular matrix to activate macrophages and neutrophils, which initiate an acute inflammatory response *via* proinflammatory cytokines such as TNF-α (56).

The high mobility groupbox-1 levels are significantly increased in infarcted brain segments (47, 57) and peripheral blood (57) in rats with early-stage CVT. There is a simultaneous increase in the proinflammatory cytokines TNF-α, IL-1β, and IL-6 (47). Pharmacological inhibition of the HMGB1 inflammatory pathway using rhs-TM (47) or glycyrrhizin, which is a natural anti-inflammatory drug (57), was found to alleviate neurological deficits and reduce the volumes of cerebral venous infarction in rats with CVT. Additionally, mechanical thrombectomy combined with glycyrrhizin significantly reduced brain injury in rats with severe CVT (57). This suggests that intravascular therapy combined with anti-inflammatory drugs may have additive or synergistic neuroprotective effects and may be a potential treatment option for severe CVT.

#### Matrix Metalloproteinase (MMPs)

Matrix metalloproteinases are a class of zinc-dependent endopeptidases extensively involved in CNS diseases, including neuroinflammation, stroke, epilepsy, and multiple sclerosis (58). Activated microglia, neutrophils, and endothelial cells can produce MMPs, which damage cerebral vessels and the BBB (26). Their gene expressions are activated by various inflammatory mediators, including TNF-α, IL-1β, IL-8, IL-17, and IL-18 (59). MMPs contribute to the neuroinflammatory pathway by acting as signaling molecules, activating the pathway, shedding death molecules, and directly hydrolyzing cerebrovascular basement membrane and tight junction proteins, which further impairs vascular integrity and ultimately causes BBB disruption and leakage (57).

Matrix metalloproteinases-9, which is among the most studied MMPs in stroke, was significantly increased in animal models of CVT (18). A recent study reported higher baseline levels of MMP-9 in patients with CVT with parenchymal brain injury than those in healthy controls (60). MMP-9 levels are associated with persistent venous occlusion, which suggests that MMP-9 is involved in brain injury resulting from severe CVT (60). However, we recently observed no significant differences in serum MMP-9 levels between patients with CVT with or without venous infarction. Additionally, none of the patients showed MMP-9 present in the cerebrospinal fluid (51). Although hypoxia increases the MMP-9 expression (61), hypoxia in venous infarction may not be as significant as that in arterial infarction, which explains the inconsistent findings. Further research is warranted to clarify the role of MMP-9 in severe CVT.

### Activation of Inflammation-Related Signaling Pathways

Various inflammatory factors can activate inflammatory pathways, which prompt the release of downstream inflammatory mediators to further aggravate inflammatory response. The classical inflammatory signaling pathways include the TLRs, NF-κB, and MAPK signaling pathways (62). These pathways are closely related to stroke and are involved in neuronal injury and cell death. The cerebral infarction volume can be reduced and neurological function can be protected by injecting the inhibitors of these pathways or knocking out the corresponding genes (63). However, the relationship between the aforementioned pathways and severe CVT remains unclear.

There has been increasing research on the NLRP3 inflammatory signaling pathway. The NLRP3 inflammasome is an intracellular protein-polymer crucially involved in innate immunity. It is present in the immune and inflammatory cells, including macrophages and monocytes, and belongs to a family of PRRs. NLRP3 inflammasome activation requires at least two signals. In the presence of immune activators, including pathogen-associated molecular patterns in pathogens and DAMPs released from injured or necrotic cells, TLRs on the inflammatory cell surface is phosphorylated and subsequently activate NF-κB. Nuclear NF-κB promotes the transcription of NLRP3, pro-IL-18, and pro-IL-1β, which are subsequently retained in the cytoplasm in an inactive form after translation. In short, activated NF-κB is the initial activation signal of the NLRP3 inflammasome (64).

Extensive DAMP stimulation promotes NLRP3 inflammasome assembly (65) by allowing the oligomerization of inactive NLRP3, apoptosis-associated speckle-like protein, and pro-Caspase-1 to activate the NLRP3 inflammasome. This complex catalyzes the conversion of pro-Caspase-1 to Caspase-1, which prompts the production and secretion of mature IL-1β and IL-18 (62). This is the second activation signal for NLRP3 inflammasome (66). These are the steps of the NLRP3-Caspase-1-IL-1β/IL-18 signaling pathway, which are involved in the progress of inflammation.

The NLRP3 inflammatory signaling pathway is crucial in neurodegenerative diseases, metabolic diseases, autoimmune diseases, atherosclerosis, and stroke (63). Animal model studies have observed significantly increased levels of NLRP3 inflammatory pathway-related molecules (NLRP3, Caspase-1, IL-1β, IL-18) in venous infarcts in the brain tissue after acute CVT (18). Similarly, Ding et al. (19) reported activation of microglia-derived NLRP3 inflammasomes after acute severe CVT. NLRP3 significantly increased from 6 h after CVT while Caspase-1 levels were significantly upregulated from day 1. Both peaked on the third day and remained at a high level until the seventh day.

The NLRP3 inflammasome mediates inflammatory neuronal death after acute severe CVT (19), also termed apoptosis (64), which may be an unrecognized mechanism of post-CVT neuronal injury. Additionally, this mode of cell death can be validated by studies on inflammation in stroke (66). Inhibiting NLRP3 can significantly downregulate downstream inflammatory molecules to reduce neuronal injury/BBB disruption and stroke severity, and therefore, improve neurological outcomes (67). Briefly, the NLRP3 inflammatory signaling pathway is a potential therapeutic target in severe CVT.

## Inflammation-Sensitive Biomarkers in Severe CVT

### High Sensitivity C-Reactive Protein (hs-CRP)/C-Reactive Protein (CRP)

C-reactive protein is a part of the innate immune response. It belongs to the acute phase proteins and is extensively synthesized by hepatocytes within a few hours after tissue injury or infection. IL-6, which is a powerful contributor to CRP production, can regulate CRP synthesis in hepatocytes at the transcriptional level, with this effect being enhanced by IL-1β. In acute inflammatory reactions of humans, plasma CRP levels rapidly increase up to 1,000-fold or more (68). As a non-specific inflammatory marker, CRP is related to the risks of arteriovenous thrombosis (68, 69) and cardiovascular disease (70). A high-sensitivity CRP (hs-CRP) assay is widely used to quantitatively determine the CRP levels (70).

There has been increasing attention on the relationship between CRP/hs-CRP and severe CVT. Clinical evidence has demonstrated significantly increased plasma CRP/hs-CRP levels in patients with severe CVT (17, 50–52, 71, 72). Notably, the hs-CRP levels are higher in the acute CVT phase than in the subacute phase, with the lowest levels being observed in the chronic phase (17, 51), which is consistent with the CRP levels. Additionally, the serum hs-CRP levels are positively correlated with baseline cerebral venous infarction, seizures, and NIH Stroke Scale scores, which suggest a significant correlation between inflammation and CVT severity (17, 51).

A recent study suggested that high CRP levels at baseline may be a novel predictive marker of poor functional prognosis at 3 months in patients with CVT (50). Among patients with acute/subacute CVT, patients with cerebral venous infarction have dramatically higher serum hs-CRP levels than those without (51). These findings suggest that the hs-CRP/CRP levels may be an assessment factor in the course of severe CVT.

### Thrombo-Inflammatory Biomarkers

The platelet-to-lymphocyte ratio (PLR) and neutrophil-to-lymphocyte ratio (NLR) are accessible laboratory parameters in clinical practice. They provide information regarding primary hemostasis and inflammation; moreover, there has been increasing attention to their utility in assessing the risk of arteriovenous thrombotic events (73). Single-center clinical studies have reported significantly higher levels of PLR (74) and NLR (17, 42, 74) in patients with acute CVT than in patients with chronic CVT. Notably, the NLR (16, 17, 74) and PLR levels (74) are positively correlated with the baseline disability degree, with NLR being more commonly used to predict adverse outcomes in patients with severe CVT. Numerous studies have demonstrated that increased NLR levels are associated with poor prognosis in patients with CVT. Taken together, increased NLR levels on admission are predictive of poor long-term prognosis in patients with CVT (17, 50, 75).

The Systemic Immune Inflammation Index (SII) is a novel cellular immune inflammation marker that is calculated as follows: platelet (/L) × neutrophil (/L)/lymphocyte (/L). Similar to NLR and PLR, it is calculated from whole blood; therefore, it is a simple and stable parameter. Recent studies have used SII as an inflammation and prognosis marker in clinical diseases (76). In patients with acute/subacute CVT, the SII values are positively correlated with mortality. SII is an independent prognostic factor for patients with non-chronic CVT, with high SII values on admission suggesting poor outcomes (75, 77). Moreover, SII and NLR were positively correlated with CVT severity (74).

The lymphocyte-to-monocyte ratio (LMR), which also serves as a ratio index, has also been explored in studies on severe CVT. LMR is a blood marker that reflects the balance between lymphocytes and monocytes, which represents baseline inflammation and immune status. Decreased and increased levels of lymphocytes and monocytes, respectively, are associated with poor outcomes in patients with stroke. Specifically, the LMR is negatively associated with stroke severity (78). Several retrospective studies have demonstrated that low LMR levels on admission are associated with adverse outcomes in patients with severe CVT (79); moreover, the LMR levels gradually increase as the disease resolves (42). The aforementioned ratio indexes are simple, low-cost, and easy to obtain; additionally, they offer significant prognostic information regarding patients with CVT.

## Anti-Inflammation Therapy: A Potential Target for Treating Severe CVT

The discussed findings suggest that inflammation is crucially involved in the pathophysiology of severe CVT. Currently, treatment strategies for severe CVT mainly include anticoagulant therapy, endovascular therapy, and symptomatic treatment. Standardized anticoagulant therapy is internationally recognized as the primary treatment for CVT and the basis for combining other treatments (2). However, current anticoagulant therapies for severe CVT cannot selectively inhibit inflammation. Therefore, further studies are warranted on anti-inflammatory treatments as a promising therapeutic option for severe CVT.

A study using murine models reported that CVT-induced brain edema and BBB dysfunction can be significantly reduced by inhibiting neutrophil infiltration (e.g., using CD18 monoclonal antibody or antineutrophil serum) (22). Rhs-TM or glycyrrhizin has a neuroprotective effect on severe CVT brain injury by inhibiting HMGB1 and its downstream proinflammatory cytokines (47, 57).

Steroids have extensive anti-inflammatory effects and decrease vasogenic edema (2). Steroid therapy is recommended in some patients with immune-inflammatory CVT associated with Behcet's disease, systemic lupus erythematosus, antiphospholipid syndrome, or Sjögren's syndrome (80–82). However, steroid therapy is not recommended for non-inflammatory CVT (2, 80). Further studies are warranted to strengthen this low-level evidence (2). We found that glucocorticoid pulse therapy combined with anticoagulation may effectively improve the prognosis of patients with acute/subacute severe CVT (83).

These findings demonstrate the potential ameliorative effect of anti-inflammatory treatments on acute/subacute severe CVT; however, further research is warranted before the anti-inflammatory treatments can be applied in clinical practice.

## Limitations and Conclusion

This is the first review to demonstrate the important role of inflammation in severe CVT. However, the types of inflammatory cells, their evolution, the cell-cell interactions, and the specific mechanism of inflammatory mediators and inflammatory pathways remain unclear. Additionally, there have been few preclinical studies on the effects of anti-inflammatory treatments on severe CVT. Our single-center case report showed that the extensive anti-inflammatory effects of glucocorticoids were efficacious against non-inflammatory severe CVT. However, further research is needed on the specific anti-inflammatory targets.

In conclusion, the inflammatory cascade is rapidly activated after the onset of severe CVT. Furthermore, there may be a mutually causal relationship between cerebral venous infarction and inflammatory response. The anti-inflammatory treatments may break the positive feedback loop between inflammation and poor prognosis to promote the recovery of patients with severe CVT. This review provides novel insight into the pathophysiology and pathogenesis of severe CVT, which could inform the development of promising anti-inflammatory therapeutic strategies for improving the prognosis of patients with severe CVT.

## Author Contributions

This manuscript was written by SH. Modifications were suggested by HL, HZ, YD, and JD critically edited the final manuscript. All authors contributed to the article and approved the submitted version.

## Funding

The study was funded by the Beijing Municipal Science and Technology Commission (Z161100000516088), Beijing Natural Science Foundation (7182064).

## Conflict of Interest

The authors declare that the research was conducted in the absence of any commercial or financial relationships that could be construed as a potential conflict of interest.

## Publisher's Note

All claims expressed in this article are solely those of the authors and do not necessarily represent those of their affiliated organizations, or those of the publisher, the editors and the reviewers. Any product that may be evaluated in this article, or claim that may be made by its manufacturer, is not guaranteed or endorsed by the publisher.
